# Muscle activation patterns during walking from transtibial amputees recorded within the residual limb-prosthetic interface

**DOI:** 10.1186/1743-0003-9-55

**Published:** 2012-08-10

**Authors:** Stephanie Huang, Daniel P Ferris

**Affiliations:** 1 Human Neuromechanics Laboratory, University of Michigan, 401 Washtenaw Ave, Ann Arbor, MI, 48109–2214, USA; 2 Department of Biomedical Engineering, University of Michigan, 401 Washtenaw Ave, Ann Arbor, MI, 48109-2214, USA; 3 School of Kinesiology, University of Michigan, 401 Washtenaw Ave, Ann Arbor, MI, 48109-2214, USA

**Keywords:** Amputee, Gait, Rehabilitation, Prosthesis, Electromyography

## Abstract

**Background:**

Powered lower limb prostheses could be more functional if they had access to feedforward control signals from the user’s nervous system. Myoelectric signals are one potential control source. The purpose of this study was to determine if muscle activation signals could be recorded from residual lower limb muscles within the prosthetic socket-limb interface during walking.

**Methods:**

We recorded surface electromyography from three lower leg muscles (*tibilias anterior*, *gastrocnemius medial head*, *gastrocnemius lateral head*) and four upper leg muscles (*vastus lateralis*, *rectus femoris*, *biceps femoris*, and *gluteus medius*) of 12 unilateral transtibial amputee subjects and 12 non-amputee subjects during treadmill walking at 0.7, 1.0, 1.3, and 1.6 m/s. Muscle signals were recorded from the amputated leg of amputee subjects and the right leg of control subjects. For amputee subjects, lower leg muscle signals were recorded from within the limb-socket interface and from muscles above the knee. We quantified differences in the muscle activation profile between amputee and control groups during treadmill walking using cross-correlation analyses. We also assessed the step-to-step inter-subject variability of these profiles by calculating variance-to-signal ratios.

**Results:**

We found that amputee subjects demonstrated reliable muscle recruitment signals from residual lower leg muscles recorded within the prosthetic socket during walking, which were locked to particular phases of the gait cycle. However, muscle activation profile variability was higher for amputee subjects than for control subjects.

**Conclusion:**

Robotic lower limb prostheses could use myoelectric signals recorded from surface electrodes within the socket-limb interface to derive feedforward commands from the amputee’s nervous system.

## Introduction

Recent advances in robotic technology have allowed for the development of powered lower limb prostheses that improve ambulation for amputees. A major feature of these new devices is the ability to interject mechanical power into the gait cycle to replace the mechanical power that is lost due to missing biological muscles. Hugh Herr’s research group at the Massachusetts’s Institute of Technology has developed a robotic ankle that uses a finite state controller to modulate ankle dynamics during gait and add power to the trailing limb during push off [1-3]. The prosthesis uses intrinsic sensing of kinetics and kinematics (e.g., heel- and toe-contact, ankle angle, and ankle torque) to determine when to transition between gait phases during walking. Their powered prosthesis resulted in lower metabolic cost compared to traditional passive elastic prostheses for level ground walking [4]. In addition to a robotic ankle, they have developed a variable impedance robotic knee that uses intrinsic sensing and a finite state controller to modulate knee stiffness during level ground walking [5]. Michael Goldfarb’s research group at Vanderbilt University has developed a robotic knee and ankle for transfemoral amputees that also uses intrinsic sensing and finite state control [6-8]. Tom Sugar’s research group at Arizona State University developed a powered ankle that relies on elastic elements to store energy and amplify mechanical power generated by the actuator [9]. It uses intrinsic sensing to detect heel strike and then the controller initiates a predetermined gait pattern. This sampling of robotic prostheses is representative of the intrinsic sensing approaches that are beginning to be utilized for prosthetic control [10,11].

There are advantages and disadvantages of controlling prosthetic lower limbs via intrinsic sensing. An advantage of prosthetics that rely on kinetic and kinematic sensing to infer user intent is that all of the sensors and associated computational hardware are built directly into the prosthetic. The interface with the human is purely mechanical, which simplifies socket design. These prosthetics generally have low step-to-step variability due to the robustness of the finite state controllers and the low sensor noise. Controllers based on intrinsic sensing tend to work well for stereotyped or cyclical tasks, such as gait. One of the inherent drawbacks of these devices is that control based on intrinsic sensing is not very good at aperiodic or highly variable motor tasks. For example, going up on the toes to reach a higher shelf would be very difficult for a state-based controller to perform using intrinsic sensing. Similarly, tasks with highly variable step-to-step kinematics such as traversing obstacles in the terrain, traversing unstable terrain, or negotiating through a crowd of people, or dealing with a variety of natural surfaces like sand and rocks would be difficult to deal with using intrinsic sensing alone.

An alternative to controllers that rely solely on intrinsic kinematic and kinetic sensing is to directly connect the prosthesis dynamics to the user’s nervous system via electromyography [12-14]. Myoelectric control has been implemented for powered upper limb prostheses. High costs have limited widespread acceptance of these devices but cost will continue to fall with continued technological advances. A more lasting obstacle to widespread acceptance of powered upper limb prostheses is the degrees of freedom that must be controlled. The human hand and wrist have more than 20 mechanical degrees of freedom but upper limb prostheses usually rely on fewer than 6 myoelectric control sources. This limits the ability for users to accurately and reliably control prosthesis mechanics. For the lower limb, fewer mechanical degrees of freedom are necessary to provide functional motor ability. For a transtibial amputee, active mechanical plantar flexion/dorsiflexion and passive foot elasticity can provide a huge energetic improvement compared to passive lower limb prostheses [4].

Controlling a limited number of mechanical degrees of freedom with myoelectric signals is feasible. Transfemoral amputees can learn to volitionally control virtual knee/ankle joint movements using myoelectric control signals from residual thigh muscles while seated and not wearing their prosthesis [15,16]. In addition, transtibial amputees can learn to volitionally activate residual muscles during the swing phase of walking to switch between level-ground walking and stair-descent locomotion modes [1]. To the best of our knowledge, this is the only case where myoelectric signals have been recorded from within the socket-limb interface during walking and used for user movement intent recognition.

To implement more robust myoelectric controllers for transtibial prostheses, it is important to assess lower leg electromyographic signal quality, variability, and adaptability during amputee gait. In the near future, it may be possible to use intramuscular electromyography sensors (IMES) to transmit electromyographic signals through the socket interface without breaking the skin [17-19]. These IMES would make it feasible to implement a wide range of myoelectric control methods with powered prostheses. However, rather than waiting for these IMES to be approved for human testing, we have recorded electromyography from lower leg muscles of transtibial amputees within the socket interface using surface electrodes. The purposes of this study were 1) to determine if surface electromyography signals can be recorded from residual lower leg muscles inside the prosthetic socket during walking, and 2) to quantify differences in muscle activation patterns between amputee and non-amputee subjects during walking.

## Methods

### Subjects

We recruited twelve unilateral transtibial amputee subjects (10 male, 2 female; age = 46 ± 18 yrs.; height = 175 ± 8 cm.; mass = 81 ± 10 kg.; mean ± s.d.) and twelve non-amputee subjects (8 male, 4 female; age = 37 ± 15 yrs.; height = 173 ± 15 cm.; mass = 76 ± 18 kg.) to participate in this study. All subjects were free of musculoskeletal and cardiovascular conditions that would limit their ability to walk safely on a treadmill. All amputee subjects had been using their prosthesis for at least six months, were accustomed to walking on their prosthesis all day, and could walk comfortably without the use of an additional ambulatory aid. Amputee subject details are provided in Table 1.

**Table 1:** Amputee subject details

**Subject**	**Reason**	**Age (yrs.)**	**Post-Amputation (yrs.)**
A01	Cancer	20	11
A02	Trauma	49	7
A03	Cancer	18	6
A04	Trauma	66	7
A05	Trauma	31	1
A06	Trauma	55	1
A07	Trauma	56	40
A08	Trauma	44	5
A09	Dysvascular	65	10
A10	Trauma	61	41
A11	Trauma	59	8
A12	Trauma	27	3

### Instrumentation

**Figure 1:**
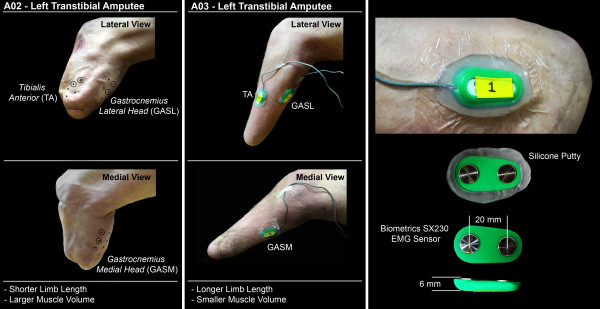
**Surface electrode placement for residual lower leg muscles.***Tibialis Anterior* (TA), *Gastrocnemius Medial Head* (GASM), *Gastrocnemius Lateral Head* (GASL). Two amputee subjects (*A02*, *A03*) show the extent of variation in lower leg shape of our amputee subjects. Subject *A02* (49 year old, amputation due to trauma at age 42) has a relatively short lower leg with relatively large muscle volume. In comparison, subject *A03* (18 year old, amputation due to cancer at age 12) has a longer lower leg with smaller muscle volume. As shown on subject *A02*, a grid of potential electrode locations was marked on the skin surface over the lower leg TA, GASM, and GASL. From each grid, the primary electrode site was determined by palpation during voluntary contractions of the muscle. Electrodes were placed over the primary electrode site and the gel liner and socket were worn over the electrodes. No modifications to the gel liner or socket were made. Socks of varying thickness were used to adjust socket-fit. Subjects were asked to walk around the laboratory to assess comfort at the primary electrode sites. If there was discomfort, electrodes were repositioned slightly or secondary sites were selected. The final electrode sites for subject *A02* are circled. After the electrode sites were finalized, silicone putty was placed around the electrode and the electrode was secured to the skin using a piece of Tegaderm^TM^ dressing.

We collected surface electromyography (EMG) from seven lower limb muscles: *tibialis anterior*, *gastrocnemius medial head*, *gastrocnemius lateral head*, *vastus lateralis*, *rectus femoris*, *biceps femoris*, and *gluteus medius*. We recorded EMG signals at 1000 Hz using pre-amplifier electrodes (Biometrics Ltd, SX230) from the amputated leg of amputee subjects and the right leg of non-amputee subjects. For upper leg muscles of all subjects and lower leg muscles of control subjects, we placed the electrode over the muscle belly and along the direction of the muscle fibers. To determine the location an orientation of each electrode, we palpated each muscle area while subjects performed a series of voluntary muscle activations. For the lower leg muscles (*tibialis anterior*, *gastrocnemii*) of amputee subjects, we marked a grid of potential recording sites on the skin surface over each muscle that we identified by palpating underlying tissue and bone. We avoided sensitive skin areas and bony protuberances. We subjectively ranked each recording site on the grid based on muscle quality (perceived by palpating the muscle area during voluntary muscle activations). We positioned one electrode over the “best” recording site on each muscle and subjects donned their prosthesis and walked around the laboratory to assess comfort. We did not make any modifications to their prosthesis. To adjust socket fit, subjects changed the thickness of socks they wore between the gel liner and prosthesis socket. If subjects expressed discomfort with an electrode, we shifted the position slightly or chose a secondary recording site. Once the recording sites were finalized, we placed silicone putty around the edges of the electrodes and secured the electrodes to the skin using Tegaderm^TM^ dressing. The silicon putty minimized skin irritation around the electrode edges. The sensor placement procedure is outlined in Figure 1. We placed the ground electrode on the *lateral malleolus* of the intact leg for amputee subjects and the *lateral malleolus* of the right leg for non-amputee subjects.

We recorded ground reaction forces in the vertical, medial-lateral, and fore-aft directions at 1000 Hz using a custom-built instrumented split-belt treadmill [20]. We defined heel-strike and toe-off events from vertical ground reaction force.

### Protocol

The first part of the test protocol assessed the subject’s ability to differentiate plantar flexor and dorsiflexor muscle activation. Subjects performed maximum voluntary activation trials where they tried to isolate the activation of their *tibialis anterior* (dorsiflexion trial) and *gastrocnemii* (plantar flexion trial) muscles. Subjects were seated upright on a raised platform so that their feet did not contact the ground during the maximum voluntary activation trials. To obtain maximal activation of the *tibialis anterior*, we instructed subjects to point their feet and toes towards the ceiling as hard as possible and sustain muscle activation at maximum dorsiflexion. To obtain maximal activation of the *gastrocnemii*, we instructed subjects to point their feet and toes towards the ground as hard as possible and sustain muscle activation at maximum plantar flexion. All ankle movements were performed bilaterally. We instructed amputee subjects to activate their lower leg muscles as if they had an intact ankle and foot. During practice trials, we displayed real time EMG signals to amputee subjects to provide feedback on the level of muscle activation. Once EMG signals appeared consistent, we recorded three repetitions for each maximum voluntary activation task. For each repetition, we asked the subjects to sustain the maximum voluntary activation for five seconds then rest with muscles fully relaxed for five seconds.

The second part of the test protocol assessed muscle activation patterns during walking. Subjects walked on a treadmill at four speeds (0.7, 1.0, 1.3, and 1.6 m/s) for two minutes at each speed. Not all subjects were able to walk at the two faster speeds. To determine the fastest walking trial that subjects could complete safely, we asked each subject to practice walking on the treadmill starting at the slowest speed. If they could walk comfortably at the given speed, we increased the treadmill speed gradually to the next level. We continued this until the fastest treadmill speed was reached or until the subject could no longer maintain walking speed. All subjects completed the 0.7 and 1.0 m/s trials. Eight of the twelve amputee subjects and eleven of the twelve control subjects completed the 1.3 m/s trial. Seven of the twelve amputee subjects and eleven of the twelve control subjects completed the 1.6 m/s trial.

### Signal processing

We performed all signal processing and statistical analyses using the R computing environment (R Development Core Team, 1999). We processed EMG signals using two separate methods. To look at raw EMG, we applied a high-pass filter (bidirectional Butterworth, 4^th^ order, 50 Hz cutoff frequency) and then demeaned the signal. We chose a cutoff frequency of 50 Hz to ensure that motion artifacts were attenuated. To analyze the frequency content of the signal, we calculated a smoothed periodogram estimated by a discrete Fourier transform and filtered using Daniell smoothers (single span of length 5). We calculated an empirical cumulative distribution function of the power spectrum to compare the distribution of frequency content between the amputee and control groups.

For the maximum voluntary activation trials, we performed frequency analysis of the *tibilias anterior* and *gastrocnemii* EMG for two seconds of sustained activation. For each subject, we selected the repetition where the maximum amplitude of the rectified signal (high-pass filtered and demeaned) was the greatest across trials. For some amputee subjects, the residual limb *tibialis anterior* was activated more than the *gastrocnemii* during the plantar flexion trial and vice versa during the dorsiflexion trial. For 1.0 m/s walking, we performed frequency analysis of the *tibilias anterior* and *gastrocnemii* for a single gait cycle. For each subject, we selected the gait cycle where the variance of the signal (high-pass filtered and demeaned) was closest to the mean variance of all cycles.

To quantify muscle activation profiles, we calculated EMG intensity using a wavelet decomposition method [21]. We calculated an intensity curve by summing across wavelets 4 (center frequency = 62.1 Hz) through 11 (center frequency = 395.5 Hz) in time. This method was chosen over other methods (e.g. generating a linear envelope using a low-pass filter) because the intensity curve provided a more distinct profile, specifically at transitions between baseline and activation. We divided the intensity curve into cycles defined by consecutive heel strike events. We normalized time by interpolating over 500 equally spaced points per cycle using cubic splines, and we normalized the amplitude to the maximum amplitude across all walking speeds. We calculated a mean intensity curve from 40 consecutive time- and amplitude-normalized cycles. To quantify the repeatability of the recorded EMG signals, we calculated a variance-to-signal ratio (VSR) as the sum of the signal variance over the sum of the signal mean squared across the 40 consecutive normalized intensity curves: $VSR=\frac{\Sigma_{i=1}^{500}\sigma_{i}^{2}}{\Sigma_{i=1}^{500}\mu_{i}^{2}}$[22]. To quantify differences in EMG shape, we used mean intensity curves to calculate normalized cross-correlations with zero time lag [23] between: 1) control group grand mean and control subject mean $\rho_{\bar{X}X_{i}}$, 2) control group grand mean and amputee subject mean $\rho_{\bar{X}Y_{i}}$, and 3) amputee group grand mean and amputee subject mean $\rho_{\bar{Y}Y_{i}}$. For cross-correlations $\rho_{\bar{X}X_{i}}$ and $\rho_{\bar{Y}Y_{i}}$, individual subject data was excluded from the group mean. Normalized cross-correlations were calculated for EMG from all seven muscles using the subset of subjects who completed all four walking speeds.

### Statistical analyses

We performed two separate ANOVAs to determine if there were significant differences in median EMG frequency between subject groups during either maximum voluntary activations or treadmill walking at 1.0 m/s. (model: *median frequency* ~ *muscle + group*). We performed another ANOVA to determine if there were significant differences in median EMG frequency between maximum voluntary activation and treadmill walking (factor: *task*) at 1.0 m/s for lower leg muscles only (model: median frequency ~ muscle + group*task). We performed two ANOVAs to determine if there were significant differences in cross-correlation (*R*-value) between subject groups (model: *R*-value ~ *muscle* + *group*). For the first ANOVA, the independent variable was $\rho_{\bar{X}X_{i}}$ for control subjects and $\rho_{\bar{X}Y_{i}}$ for amputee subjects. For the second ANOVA, the independent variable was $\rho_{\bar{X}X_{i}}$ for control subjects and $\rho_{\bar{Y}Y_{i}}$ for amputee subjects. For all ANOVAs, if factors of interest were significant (p < 0.05), we performed a Tukey’s Honestly Significant Difference test to determine which contrasts were significant (p < 0.05).

## Results

### Maximum voluntary activation of lower Leg muscles

**Figure 2:**
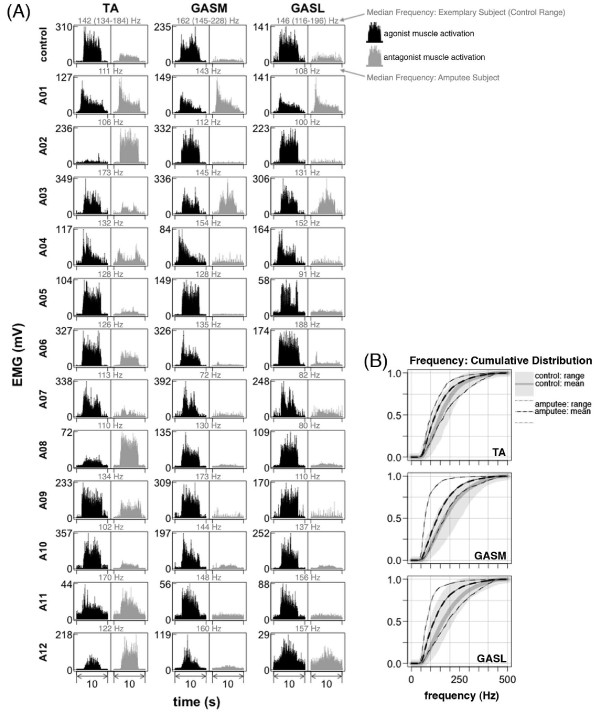
**Lower leg EMG maximum voluntary activation.***Tibialis Anterior* (TA), *Gastrocnemius Medial Head* (GASM), *Gastrocnemius Lateral Head* (GASL). (**A**) EMG during maximum voluntary activation of the *tibialis anterior* and *gastrocnemii* muscles during seated dorsiflexion and plantar flexion. Data is shown for one exemplary control subject and twelve amputee subjects. Signals are high-pass filtered, demeaned, and rectified (for visualization). Signals in black indicate that the muscle is expected to act as an agonist to the ankle movement. Signals in gray indicate that the muscle is expected to act as an antagonist to the ankle movement. Median frequency during maximum voluntary activation (agonist or antagonist depending on which activation had the greatest amplitude) is shown above each plot in gray. In control subjects, there was high agonist muscle activation (black) and low antagonist muscle activation (gray). This activation pattern was not consistent in amputee subjects. Amputee subjects *A02* and *A08* had little to no lower leg muscle activation during dorsiflexion and high activation of both the *tibialis anterior* and *gastrocnemii* muscles during plantar flexion. *A01* had activation of all lower leg muscles for both dorsiflexion and plantar flexion, but the activation level was not well sustained. Some amputee subjects had activation patterns similar to controls (*A05*, *A06*, *A07*, *A09*, *A10*). (**B**) Empirical cumulative density function of EMG power spectrum. Lines are shown for group means and boundaries indicate group range.

Amputee subjects were able to volitionally activate their lower leg muscles during the maximum voluntary activation trials but the relative activation of agonist and antagonist muscles was not consistent across subjects (Figure 2A). All control subjects had high and well-sustained agonist muscle activation and low antagonist muscle activation during the trials. Some amputee subjects had muscle activation patterns similar to controls (e.g., Figure 2A, subjects *A05*, *A06*, *A07*, *A09*, and *A10*). These subjects had a range of 1–41 years since amputation (Table 1). A couple of amputee subjects had high activation of both agonist and antagonist muscles during plantar flexion and little to no activation of agonist or antagonist muscles during dorsiflexion (e.g., Figure 2A, subjects *A02* and *A08*). Although most amputee subjects were able to sustain activation levels as well as control subjects, some had difficulty maintaining activation levels (e.g., Figure 2A, subjects *A01* and *A04*).

### Lower Leg EMG during walking

**Figure 3:**
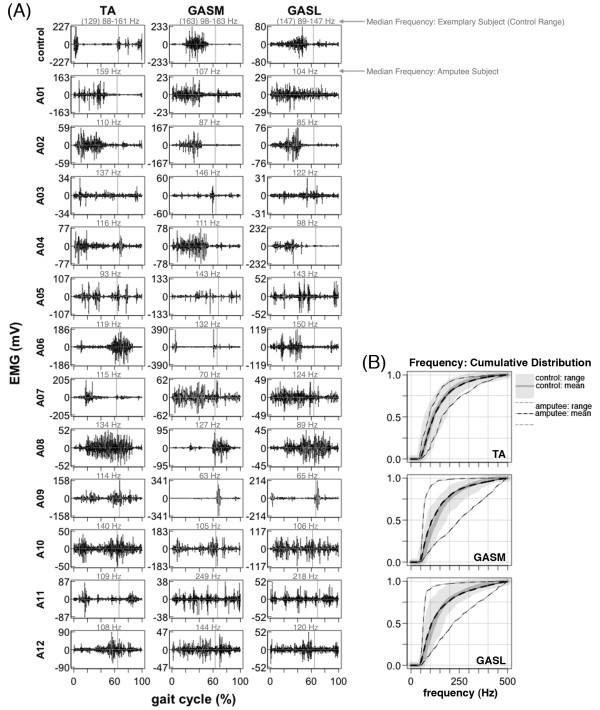
**Lower leg EMG activation during 1.0 m/s walking.***Tibialis Anterior* (TA), *Gastrocnemius Medial Head* (GASM), *Gastrocnemius Lateral Head* (GASL). (**A**) Raw EMG signals from the *tibialis anterior* and *gastrocnemii* muscles for a single stride (1.0 m/s). Data is shown for one exemplary control subject and twelve amputee subjects. EMG signals are high-pass filtered and demeaned. Vertical lines show toe-off. Median frequency is shown above each plot in gray. There was a lot of variability in EMG signal patterns across amputee subjects. Amputee subject *A11* (GASM, GASL) had several EMG bursts that were approximately equally spaced and of similar amplitude across the gait cycle. A similar pattern was seen in *A10* (GASL) and *A05* (TA). Amputee subject *A09* (GASM, GASL) had short EMG bursts of high amplitude that occurred shortly after toe-off. A similar pattern was seen in *A06* (GASM) with two high-amplitude EMG bursts that occurred shortly after heel-strike and shorty before toe-off. In both *A06* and *A09,* the amplitude of the EMG bursts exceeded those recorded during maximum activation trials. (**B**) Empirical cumulative density function of EMG power spectrum. Lines are shown for group means and boundaries indicate group range.

During treadmill walking, *tibialis anterior*, *gastrocnemius medial head*, and *gastrocnemius lateral head* activation patterns in amputee subjects had much higher inter-subject variability and were substantially different than the patterns of the control subjects (Figure 3A, Figure 5A, Figure 7). The high inter-subject variability in amputee EMG patterns is demonstrated by a significant difference (ANOVA, p < 0.001) in EMG pattern cross-correlation between the amputee individual data vs. amputee mean, compared to the control individual data vs. the control mean $\rho_{\bar{Y}Y_{i}},\rho_{\bar{X}X_{i}}$ (Table 2)*.* Mean cross-correlations for individual amputee EMG patterns vs. the amputee mean $\rho_{\bar{Y}Y_{i}}$ ranged from 0.20-0.53 for the *tibialis anterior*, *gastrocnemius medial head*, and *gastrocnemius lateral head* (Table 2). In comparison, mean cross-correlations for individual control EMG patterns vs. the control mean $\rho_{\bar{X}X_{i}}$ ranged from 0.73-0.92 for the same muscles (Table 2). In addition to the difference in inter-subject variability, the cross-correlations also provide evidence of the difference in shape of the EMG activation patterns between amputee and control subjects. There was a significant difference (ANOVA, p < 0.001) in EMG pattern cross-correlation between the amputee individual data vs. control mean, compared to the control individual data vs. control mean $\rho_{\bar{X}Y_{i}},\rho_{\bar{X}X_{i}}$ (Table 2)*.* In the amputee group, mean cross-correlations against the control mean $\rho_{\bar{X}Y_{i}}$ ranged from −0.33 to 0.48 for the *tibialis anterior*, *gastrocnemius medial head*, and *gastrocnemius lateral head*. In the control group, mean cross-correlation against the control mean $\rho_{\bar{X}X_{i}}$ ranged from 0.73-0.92 for the same muscles.

**Figure 4:**
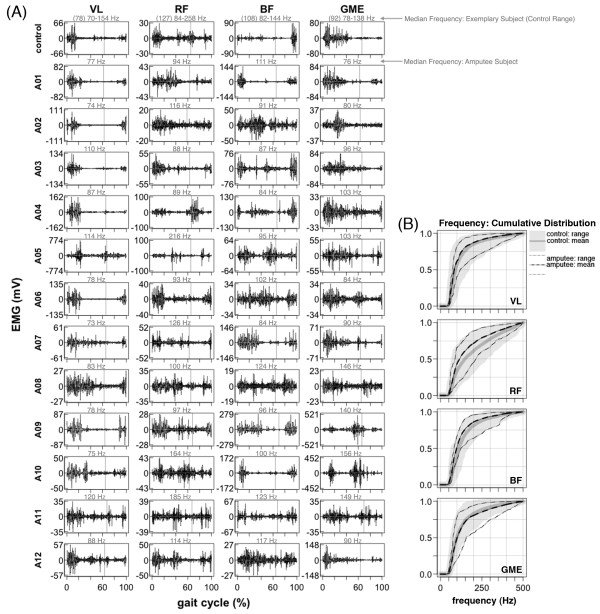
**Lower leg EMG activation profiles during 1.0 m/s walking.***Tibialis Anterior* (TA), *Gastrocnemius Medial Head* (GASM), *Gastrocnemius Lateral Head* (GASL). (**A**) Normalized mean EMG intensity curves for the *tibialis anterior* and *gastrocnemii* muscles calculated from forty consecutive strides (1.0 m/s). Control data is the grand mean of twelve control subjects. Maximum mean EMG intensity across the gait cycle is 1.0. One standard deviation above the mean is shown in gray. Vertical lines show average toe-off. Variance-to-signal ratio is shown above each plot in gray. (**B**) Variance-to-signal ratio of lower leg muscles calculated from 40 consecutive cycles at 1.0 m/s.

**Figure 5:**
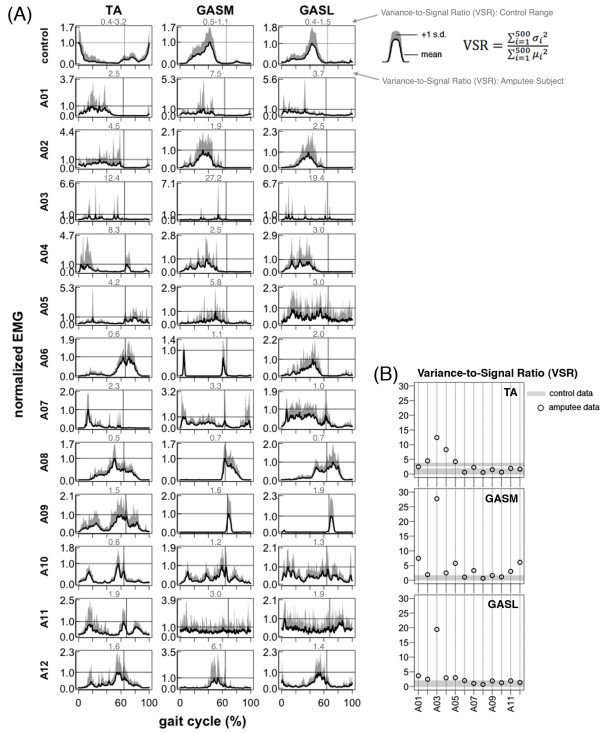
**Lower leg EMG activation profiles during 1.0 m/s walking.***Tibialis Anterior* (TA), *Gastrocnemius Medial Head* (GASM), *Gastrocnemius Lateral Head* (GASL). Mean EMG intensity curves of lower leg muscles for control group and seven amputee subjects during 0.7, 1.0, 1.3, and 1.6 m/s treadmill walking. Mean curves are calculated from 40 consecutive cycles. The grand mean curve is shown for the control group. Vertical lines show average toe-off events for the fastest and slowest walking speeds. In amputee subjects, the trend of increasing EMG amplitude with walking speed was not seen across amputee subjects. In amputee subject *A02*, the TA amplitude at 80-100% gait cycle scaled with speed and the GASM/GASL amplitude decreased with speed from 0.7-1.3 m/s then increased at 1.6 m/s. In subject *A07*, the TA at 0-20% gait cycle had relatively low activation higher speeds and high activation at 0.7-1.0 m/s. A similar pattern was seen in A12 with very high activation of the TA at 20-40% gait cycle at the slowest speed and relatively low activation at 0.7-1.3 m/s. In subject *A11*, the GASM/GASL at 0-20% of the gait cycle had relatively low activation at 0.7-1.3 m/s, but had large increase in amplitude at 1.6 m/s. In subject *A12*, there was a phase shift and increase in amplitude with speed for the TA and GASM/GASL at 40-60% gait cycle.

**Table 2:** EMG activation pattern cross-correlations

0.7 m/s	$\rho_{\bar{X}X_{i}}$	$\rho_{\bar{X}Y_{i}}$	$\rho_{\bar{Y}Y_{i}}$
	mean (sd)	mean (sd)	mean (sd)
*Tibialis Anterior*	0.73 (0.11) *°	−0.33 (0.13) °	0.44 (0.21) *
*Gastrocnemius Medial Head*	0.90 (0.09) *°	0.48 (0.42) °	0.45 (0.32) *
*Gastrocnemius Lateral Head*	0.79 (0.17) *°	0.37 (0.40) °	0.37 (0.35) *
*Vastus Lateralis*	0.81 (0.19)	0.83 (0.08)	0.89 (0.07)
*Rectus Femoris*	0.63 (0.28)	0.70 (0.23)	0.71 (0.18)
*Biceps Femoris*	0.75 (0.10) *°	0.31 (0.48) °	0.35 (0.36) *
*Gluteus Medius*	0.72 (0.31) *	0.66 (0.32)	0.67 (0.28) *
1.0 m/s	$\rho_{\bar{X}X_{i}}$	$\rho_{\bar{X}Y_{i}}$	$\rho_{\bar{Y}Y_{i}}$
	mean (sd)	mean (sd)	mean (sd)
*Tibialis Anterior*	0.80 (0.09) *°	−0.24 (0.08) °	0.32 (0.25) *
*Gastrocnemius Medial Head*	0.87 (0.08) *°	0.23 (0.40) °	0.20 (0.19) *
*Gastrocnemius Lateral Head*	0.83 (0.12) *°	0.20 (0.37) °	0.24 (0.37) *
*Vastus Lateralis*	0.86 (0.08)	0.83 (0.10)	0.90 (0.06)
*Rectus Femoris*	0.77 (0.16)	0.70 (0.23)	0.70 (0.23)
*Biceps Femoris*	0.86 (0.06) *°	0.38 (0.39) °	0.53 (0.30) *
*Gluteus Medius*	0.82 (0.14) *	0.63 (0.36)	0.61 (0.32) *
1.3 m/s	$\rho_{\bar{X}X_{i}}$	$\rho_{\bar{X}Y_{i}}$	$\rho_{\bar{Y}Y_{i}}$
	mean (sd)	mean (sd)	mean (sd)
*Tibialis Anterior*	0.84 (0.08) *°	−0.09 (0.18) °	0.20 (0.22) *
*Gastrocnemius Medial Head*	0.88 (0.07) *°	0.32 (0.26) °	0.41 (0.24) *
*Gastrocnemius Lateral Head*	0.92 (0.07) *°	0.32 (0.26) °	0.22 (0.32) *
*Vastus Lateralis*	0.89 (0.05)	0.84 (0.11)	0.84 (0.11)
*Rectus Femoris*	0.82 (0.15)	0.70 (0.23)	0.70 (0.23)
*Biceps Femoris*	0.89 (0.05) *°	0.33 (0.34) °	0.55 (0.26) *
*Gluteus Medius*	0.77 (0.19) *	0.63 (0.38)	0.56 (0.35) *
1.6 m/s	$\rho_{\bar{X}X_{i}}$	$\rho_{\bar{X}Y_{i}}$	$\rho_{\bar{Y}Y_{i}}$
	mean (sd)	mean (sd)	mean (sd)
*Tibialis Anterior*	0.85 (0.07) *°	−0.05 (0.36) °	0.27 (0.28) *
*Gastrocnemius Medial Head*	0.88 (0.07) *°	0.48 (0.28) °	0.53 (0.15) *
*Gastrocnemius Lateral Head*	0.91 (0.09) *°	0.40 (0.40) °	0.46 (0.34) *
*Vastus Lateralis*	0.90 (0.06)	0.77 (0.20)	0.74 (0.15)
*Rectus Femoris*	0.74 (0.15)	0.58 (0.30)	0.66 (0.33)
*Biceps Femoris*	0.89 (0.07) *°	0.31 (0.26) °	0.72 (0.13) *
*Gluteus Medius*	0.75 (0.18) *	0.50 (0.40)	0.45 (0.30) *

### Upper Leg EMG during walking

**Figure 6:**
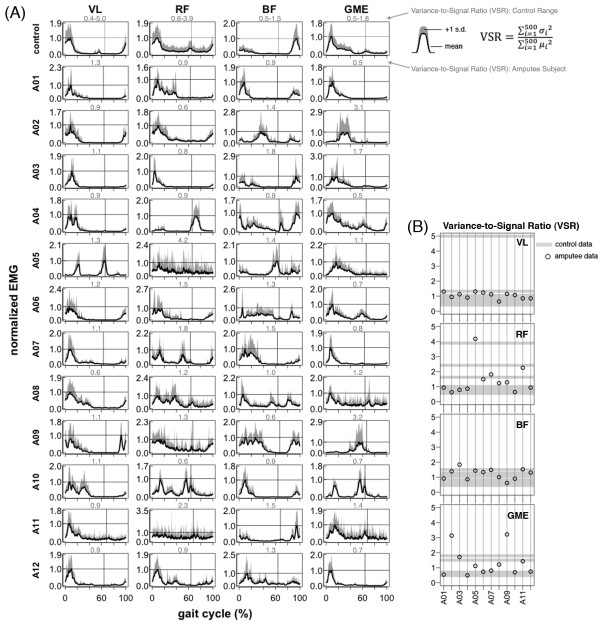
**Upper leg EMG activation during walking.***Vastus Lateralis* (VL), *Rectus Femoris* (RF), *Biceps Femoris* (BF), *Gluteus Medius* (GME). Raw EMG signals from the *vastus lateralis*, *rectus femoris*, *biceps femoris*, *and gluteus medius* muscles for a single stride (1.0 m/s). Data is shown for one exemplary control subject and twelve amputee subjects. EMG signals are high-pass filtered and demeaned. Vertical lines show toe-off. Median frequency is shown above each plot in gray. Many EMG patterns of amputee subjects are different from the control and there is a large amount of variability in EMG patterns across amputees. (**B**) Empirical cumulative density function of EMG power spectrum. Lines are shown for group means and boundaries indicate group range.

Compared to lower leg muscles, upper leg muscle activation patterns during walking were more similar between amputee and control subjects (Figure 4A, Figure 6A, Figure 8). There was no significant difference in inter-subject variability between amputees and controls for the *vastus lateralis* and *rectus femoris*$\rho_{\bar{Y}Y_{i}},\rho_{\bar{X}X_{i}}$; post-hoc *t*-test p > 0.05) (Table 2)*.* Mean cross-correlation for individual amputee EMG patterns vs. the amputee mean $\rho_{\bar{Y}Y_{i}}$ for these muscles ranged from 0.66-0.90 (Table 2). In comparison, mean cross-correlation for individual control EMG patterns vs. the control mean $\rho_{\bar{X}X_{i}}$ ranged from 0.63-0.90 for the same muscles (Table 2). For the *biceps femoris* and *gluteus medius*, there was a significant difference (post-hoc *t*-test p < 0.001) in EMG pattern cross-correlation between the amputee individual data vs. amputee mean, compared to the control individual data vs. the control mean $\rho_{\bar{Y}Y_{i}},\rho_{\bar{X}X_{i}}$. Mean cross-correlation for individual amputee EMG patterns vs. the amputee mean $\rho_{\bar{Y}Y_{i}}$ ranged from 0.35-0.72 for the *biceps femoris* and *gluteus medius*. In comparison, mean cross-correlation for individual control EMG patterns vs. the control mean $\rho_{\bar{X}X_{i}}$ ranged from 0.72- 0.89 for the same muscles (Table 2). There was no significant difference (post-hoc *t*-test p > 0.05) in EMG activation shape between amputees and controls for the *vastus lateralis*, *rectus femoris*, and *gluteus medius*$\rho_{\bar{X}Y_{i}},\rho_{\bar{X}X_{i}}$ (Table 2)*.* Mean cross-correlation for individual amputee EMG patterns vs. the amputee mean $\rho_{\bar{X}Y_{i}}$ ranged from 0.50-0.84 for the *vastus lateralis*, *rectus femoris*, and *gluteus medius* (Table 2). Mean cross-correlation for individual control EMG patterns vs. the control mean $\rho_{\bar{X}X_{i}}$ ranged from 0.63-0.90 for the same muscles (Table 2). However, the EMG activation shape for the *biceps femoris* was significantly different between the amputee subjects and the control subjects (post-hoc *t*-test p < 0.001). Mean cross-correlation for individual amputee EMG pa 8tterns against the control mean $\rho_{\bar{X}Y_{i}}$ ranged from 0.31-0.38 for the *biceps femoris* (Table 2). Mean cross-correlation for individual control EMG patterns against the control mean $\rho_{\bar{X}X_{i}}$ ranged from 0.75-0.89 for the same muscle (Table 2).

**Figure 7:**
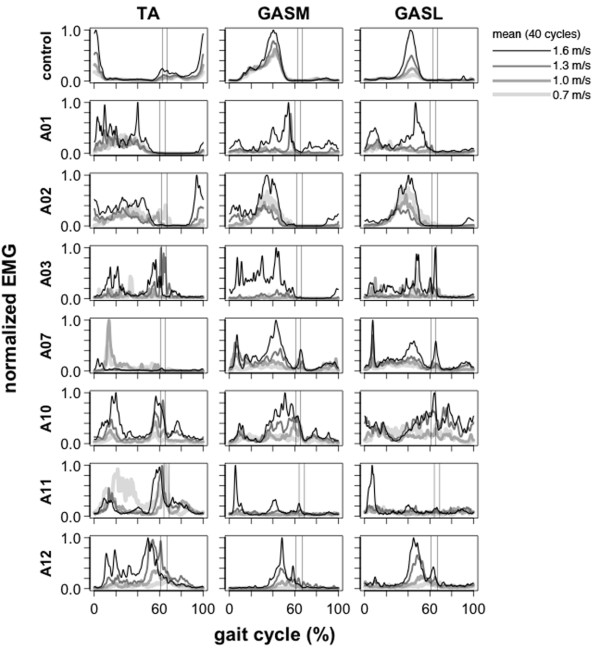
**Upper leg EMG activation profiles during walking.***Vastus Lateralis* (VL), *Rectus Femoris* (RF), *Biceps Femoris* (BF), *Gluteus Medius* (GME). (**A**) Normalized mean EMG intensity curves for the *vastus lateralis*, *rectus femoris*, *biceps femoris*, and *gluteous medius* muscles calculated from forty consecutive strides (1.0 m/s). Control data is the grand mean of twelve control subjects. Maximum mean EMG intensity across the gait cycle is 1.0. One standard deviation above the mean is shown in gray. Vertical lines show average toe-off. Variance-to-signal ratio is shown above each plot in gray. (**B**) Variance-to-signal ratio of lower leg muscles calculated from 40 consecutive cycles at 1.0 m/s.

**Figure 8:**
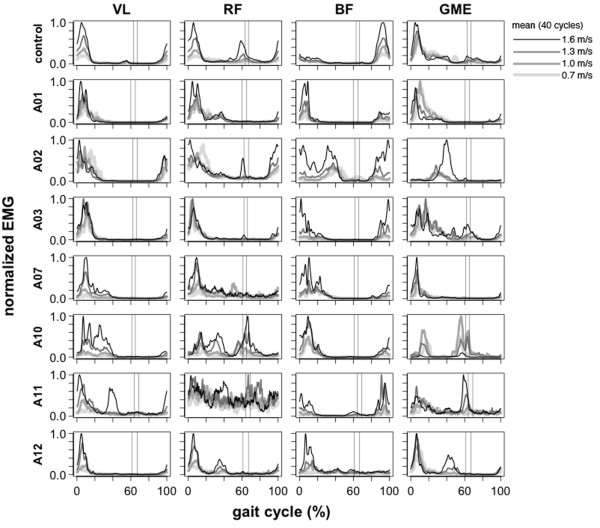
**Upper leg EMG activation profiles during walking.***Vastus Lateralis* (VL), *Rectus Femoris* (RF), *Biceps Femoris* (BF), *Gluteus Medius* (GME). Mean EMG intensity curves of upper leg muscles for control group and seven amputee subjects during 0.7, 1.0, 1.3, and 1.6 m/s treadmill walking. Mean curves are calculated from 40 consecutive cycles. The grand mean curve is shown for the control group. Vertical lines show average toe-off events for the fastest and slowest walking speeds. In amputee subjects, the trend of increasing EMG amplitude with walking speed was not seen across amputee subjects. In amputee subject *A11*, activation of the VL increased with walking speed at 0-20% of the gait cycle and also a phase shift (max activation appears to occur earlier). There was also activation of the VL around 40% of the gait cycle, but only at the fastest walking speed. There was no distinct activation pattern of the RF at any speed. There was GME activation around 60% of the gait cycle and amplitude increased with walking speed and also a phase shift (max activation appears to occur earlier). In subject *A10*, GME activation decreased with walking speed at 0-20% and 40-80% of the gait cycle. In subject *A03*, there was similar activation of the VL and RD across all walking speeds. In subject *A02*, activation of GME increased dramatically at 20-60% gait cycle for the fastest walking speed with a significant phase shift (peak activation occurs later). There was also a large increase in BF activation at the fastest walking speed.

### Inter-stride variability of EMG during walking

Variance-to-signal ratios of EMG during 1.0 m/s treadmill walking were significantly greater in the amputee group compared to the control group (control mean = 1.0, amputee mean = 2.4; ANOVA group effect, p < 0.001) (Table 3, Figures 5 and 6). However, post-hoc t-tests revealed that the only muscle with a significant difference between groups was the *gastrocnemius medial head* (post-hoc *t*-test p < 0.001).

**Table 3:** Variance-to-signal ratios for 1.0 m/s walking

	**Controls mean (sd)**	**Amputees mean (sd)**
*Tibialis Anterior*	1.0 (0.8)	3.4 (3.6)
*Gastrocnemius Medial Head*	0.8 (0.2) *	5.2 (7.4) *
*Gastrocnemius Lateral Head*	0.9 (0.3)	3.5 (5.1)
*Vastus Lateralis*	1.1 (1.2)	1.0 (0.2)
*Rectus Femoris*	1.2 (1.0)	1.4 (1.0)
*Biceps Femoris*	0.9 (0.3)	1.2 (0.4)
*Gluteus Medius*	0.7 (0.4)	1.3 (0.9)

*p < 0.001 for controls vs. amputees.

### EMG median frequencies

During maximum voluntary activation, median EMG frequencies for lower leg muscles were significantly lower in amputee subjects compared to control subjects (ANOVA group effect, p < 0.001) (Table 4, Figure 2B). However, during 1.0 m/s treadmill walking, median EMG frequencies for upper and lower leg muscles of amputee and control subjects were not significantly different (ANOVA group effect, p > 0.10) (Table 4, Figures 3 and 4). In the amputee group, median EMG frequencies of residual lower leg muscles were similar for maximum voluntary activation and 1.0 m/s treadmill walking (post-hoc *t*-test, p > 0.50) (Table 4). In the control group, median EMG frequencies of lower leg muscles were significantly greater during maximum voluntary activation compared to 1.0 m/s treadmill walking (post-hoc *t*-test, p < 0.001) (Table 4).

**Table 4:** EMG median frequencies

	**Maximum voluntary activation**		**Treadmill walking (1.0 m/s)**	
	**Controls mean (sd)**	**Amputees mean (sd)**	**Controls mean (sd)**	**Amputees mean (sd)**
*Tibialis Anterior*	153 (14) *°	127 (23) *	115 (22) °	121 (18)
*Gastrocnemius Medial Head*	174 (23) *°	137 (26) *	131 (18) °	124 (49)
*Gastrocnemius Lateral Head*	166 (23) *°	124 (34) *	122 (14) °	119 (40)
*Vastus Lateralis*			97 (24)	88 (17)
*Rectus Femoris*			156 (62)	123 (42)
*Biceps Femoris*			113 (18)	101 (15)
*Gluteus Medius*			102 (18)	109 (30)

*p < 0.001 for controls vs. amputees; °p < 0.001 for maximum voluntary activation vs. treadmill walking.

## Discussion

The main finding of this study is that during walking, most amputee subjects had residual lower leg muscle activation patterns that were entrained to the gait cycle but highly variable across subjects. The residual lower leg muscle activation patterns were very different from the normal control patterns (Figure 5). This is evidenced by the low EMG cross-correlation values between amputee subjects and the control mean for *tibialis anterior* and *gastrocnemii* (Table 2). Despite the high variability in residual lower leg EMG patterns across amputee subjects, inter-stride variability was similar to that of control subjects. The *gastrocnemius medial head* was the only muscle with a variance-to-signal ratio significantly greater in the amputee group compared to the control group. This significant difference in variance-to-signal ratio between groups was due to a single amputee subject whose variance-to-signal noise ratio was magnitudes greater than other amputee subjects (Figure 5, subject *A03*). Subject *A03* had high inter-stride variability for all three residual lower leg muscles (Figure 5). The inter-stride variability could be problematic if it continued when using a powered lower limb prosthesis under myoelectric control. However, it seems reasonable to presume that the inter-stride variability would decrease if the residual muscle activity had a functional purpose during walking (e.g., to control dynamics of a powered prosthesis). Future studies should document the variability in muscle recruitment patterns while subjects learn to use powered prostheses.

Another finding of this study is that many, but not all, amputee subjects had robust volitional control of residual lower leg muscle activation. During maximum voluntary dorsiflexion and plantar flexion, residual muscle activation profiles in several amputee subjects were similar to controls (Figure 2). The maximum activation levels were well above resting baseline, the time to reach maximum activation from resting baseline was short, and the activation levels were well sustained. Some of the amputee subjects were able to differentiate *tibialis anterior* and *gastrocnemii* activation and had coactivation levels similar to control subjects (e.g., Figure 2A, subjects *A05* and *A09*). Other amputee subjects were not able to differentiate *tibialis anterior* and *gastrocnemii* activation during volitional maximum activation. As a result, there was either complete coactivation for both plantar flexion and dorsiflexion tasks (e.g., Figure 2A, subject *A01*) or an inability to recruit any muscles strongly during dorsiflexion (e.g., Figure 2A, subjects *A02* and *A08*). For the subjects that demonstrated complete coactivation, synchronous recruitment of residual muscles was not hard-wired because their *tibialis anterior* and *gastrocnemii* activation patterns were distinctly different from each other during walking, especially at faster walking speeds (e.g., Figure 2A, subjects *A01* and *A02*). One reason that the amputee subjects may have lost robust volitional control of the residual limb muscles is the lack of proprioceptive or visual feedback of muscle activity. Without an ankle joint to provide sensory information about joint position, there is no clear information reinforcing the consequences of muscle activity. It seems likely that coupling a powered prosthetic limb to the residual limb muscle activity would increase the volitional motor control [14,24-26].

In the upper leg muscles, our data show that amputee subjects had greater inter-subject variability in their *biceps femoris* and *gluteus medius* muscle activation profiles compared to control subjects during walking (Table 2, Figure 6). In addition, our data show that amputee subjects had a different *biceps femoris* activation profile shape than control subjects (Table 2, Figure 6). Previous studies have suggested that transtibial amputees walk with greater residual leg *biceps femoris* activation during early stance compared to the intact *biceps femoris* to stabilize the knee joint [27-29] and/or increase propulsion of the residual leg [30,31]. In normal walking, the primary function of the *gluteus medius* is to provide support during early stance to midstance and the *biceps femoris* has the potential for generating support from early stance to midstance. Ankle dorsiflexors provide support during early stance and ankle plantar flexors provide support during late stance [32]. It is likely that transtibial amputees compensate for the loss of support from ankle muscles by recruiting muscles above the knee to increase walking stability during stance. The inter-subject variability in the *biceps femoris* and *gluteus medius* activation shape observed in our amputee subjects suggests that there are differences in compensatory muscle recruitment patterns used by transtibial amputees during walking.

One limitation of our study is that we did not present data from overground walking. Past studies have shown that lower limb EMG patterns and kinematics can be different during treadmill walking compared to overground walking [33,34]. Biomechanically, treadmill gait and overground gait is identical if the treadmill belt speed is constant [35]. The differences in biological gait measurements occur primarily due to two aspects: differences in visual flow [36] and treadmill speed fluctuations [37]. We did not include overground walking in this study because our primary focus was to quantify differences in signal patterns and variability between amputee and non-amputee groups and within groups. Now that we have demonstrated that reliable signals can be recorded from residual muscles of transtibial amputees during treadmill walking at constant speeds, we plan to expand our study to include lower limb EMG patterns of transtibial amputees and non-amputees during overground walking at self-selected walking speeds. This will provide a better understanding of how signals recorded from residual muscles in transtibial amputees can be utilized to control robotic lower limb prostheses. Another limitation of our study is that the mean age of our amputee group was greater than our non-amputee group. We do not believe that the results presented in this study would change significantly given more similar ages between groups, but further data could support or refute this assumption.

Several previous studies have presented EMG data from the amputated limb of transtibial amputees during walking [27,28,30,38], but they did not record EMG from residual limb muscles inside the socket. It has traditionally been thought that the mechanics of the socket-limb interface prevent reliable measurements of EMG from the residual limb muscles during walking with surface electrodes. Au et al. recorded EMG from residual limb muscles within the socket, but were only able to get a reliable signal during swing [1]. We were able to record robust and reliable EMG during both stance and swing by using active EMG electrodes to maximize signal-to-noise ratio and using silicone putty to minimize movement and discomfort at the electrode sites.

Although there was the possibility for mechanical artifacts in our EMG recordings, data of EMG median frequencies suggest that we measured muscle activity from the residual limb muscles with little to no motion artifact. The EMG median frequencies recorded from the residual limb muscles during walking were similar to the EMG median frequencies recorded from the residual limb muscles during seated maximum voluntary activation trials (Table 4). In addition, the EMG median frequencies recorded from residual lower leg muscles in amputee subjects during treadmill walking were similar to the EMG median frequencies of the intact lower leg muscles in control subjects during treadmill walking (Table 4). Some of the amputee subjects demonstrated abnormal EMG patterns that had rhythmic, short-duration, and high-amplitude bursts (e.g., Figure 3, subjects *A06* and *A09*). We do not believe that these bursts resulted from mechanical perturbations to the electrodes because of the filtering we used and the frequency content of the resulting signals. Similar EMG patterns have been demonstrated in individuals with spinal cord injury that have had long-term disuse atrophy of the muscles [39,40]. The short-duration, high-amplitude EMG bursts that occurred around heel-strike and toe-off events may have been a result of reflex activation from muscle fiber stretch (Ia and II afferents) or rapid loading/unloading (Ib afferents).

The unique residual muscle activation patterns seen in our amputee subjects during gait suggest that neural plasticity may have occurred following amputation. Previous studies have demonstrated that neural plasticity in lower limb amputees occurs predominantly at the cortical level [41,42]. Neural plasticity can be affected by cause of amputation (e.g. traumatic, cancer-related, dysvascular-related), age at amputation, surgical procedure, muscle atrophy, and degeneration of nerves. The long-term cortical reorganization that occurs following injury is also highly use-dependent [43]. Changes in gait-related muscle activity following amputation would have a major impact on use-dependent cortical plasticity. Some amputees may learn to activate their residual muscles to improve stability at the limb-socket interface or to minimize socket discomfort/pain associated with impulsive prosthetic forces. This could alter the activation patterns away from the normal functional pattern seen in intact subjects and could contribute to increased inter-subject variability in amputees.

The results of this study are encouraging for the development of powered lower limb prosthesis under myoelectric control. Coupling an amputee’s nervous system to a robotic prosthesis should provide a strong stimulus for learning to modify residual muscle activation patterns. In past studies, we have found that subjects with intact musculoskeletal systems can quickly adapt their muscle activation patterns to control powered lower-limb orthoses under proportional myoelectric control [44-47]. It seems likely that amputees could also learn to modify their muscle activation patterns to control powered lower-limb prostheses, though it may take longer due to the motor plasticity that has occurred since the amputation. Residual limb muscle activation patterns during dynamic tasks such as walking may function to improve fit and/or minimize discomfort at the socket-limb interface. Learning new residual activation patterns to control lower-limb prostheses may compete with this. Future studies should investigate why amputees adopt specific residual limb muscle activation patterns in order to assess the feasibility of myoelectric control using residual limb muscles during walking. Continued technological advances in intramuscular electrodes that could transmit control EMG signals through the prosthetic socket-limb interface without breaking the skin [17-19] would provide a means for generating feedforward control signals to a robotic prosthesis from the nervous system. Another option is recent technological advances in flexible epidermal electronics that could be mounted directly on the skin within the prosthetic socket-limb interface [48]. Either of these options could provide a long-term means for improving the control of powered lower limb prosthesis using EMG from the residual limb muscles.

## Conclusions

It is possible to record artifact-free muscle activation patterns from residual limb muscles within the prosthetic socket-limb interface with surface electromyography electrodes. There is high inter-subject variability in recruitment patterns in amputees, but for each subject EMG patterns are consistent from stride to stride. Our results support the potential use of myoelectric controllers for direct feedforward control of robotic lower limb prostheses.

## Competing interests

The authors declare that they have no competing interests.

## Authors’ contributions

SH recruited human subjects and collected data. SH processed and analyzed data, performed statistical analyses, and drafted the manuscript. DF conceived of the study and helped to draft the manuscript. DF and SH participated in the study design and contributed to interpretation of findings. Both authors read and approved the final manuscript.
